# Reintegration of Food Industry By-Products: Potential Applications

**DOI:** 10.3390/foods11223743

**Published:** 2022-11-21

**Authors:** Christos Eliopoulos, Giorgos Markou, Ioanna Langousi, Dimitrios Arapoglou

**Affiliations:** Institute of Technology of Agricultural Products, Hellenic Agricultural Organization—Demeter, L. Sof. Venizelou 1, 14123 Lykovryssi, Greece

**Keywords:** agri-food wastes, food, pharmaceuticals, cosmetics, feed

## Abstract

Numerous studies have indicated that fruits and vegetables are considered as significant sources of bioactive compounds. The generated by-products, which are derived from the food industry, reveal similar or higher antioxidant activity. On the other hand, intense industrialization results in the production of large volumes of by-products, raising serious environmental issues. Therefore, this situation creates the necessity to develop new strategies in order to exploit the generated wastes, securing the ability to develop new high-added-value products. This review aims to summarize the exploitation of fruit wastes, namely, apple and citrus, as well as vegetable by-products which are derived from tomato, potato and carrot cultivation. All the aforementioned by-products have found wide applications in the development of new high-added-value products in the food and feed industry owing to their improved nutritional profiles. Furthermore, these wastes are characterized by a strong antioxidant activity, justifying their valorization in other fields such as cosmetics and pharmaceutical industries.

## 1. Introduction

Food production has seen a significant increase worldwide, with the Food and Agriculture Organization of the United Nations (FAO) reporting that one-third of the developed products destined for human consumption (approximately 1.3 billion tonnes/year) are discarded every year [1]. Furthermore, the human population is expected to record a projected increase by 2050, indicating the necessity for the development of an appropriate food supply in order to secure the ability of food preparation for future years. In this context, population growth will contribute to a parallel increase in food waste, thus enhancing the environmental issues which are connected with its proper management and disposal [1]. Wastes’ disposal raises serious economic issues for food processors. Furthermore, the generated wastes have the potential to increase the environmental footprint by emitting CO_2_ and other greenhouse gasses [2].

The global production of municipal organic wastes is expected to exceed the amount of 1000 million tonnes per year in 2025 [3], since Europe discards 50% of its generated foods [4]. Food waste is generated using approximately 30% of the global agricultural land area [5]. The Food Waste Index Report stated that during 2019 the generated by-products were estimated at 931 million tonnes, with 61% being categorized as household wastes, 26% as food service wastes and, finally, the remaining 13% as retail wastes. Food wastes production, especially for those which are defined as household, is associated with consumers’ purchasing power depending on the respective country [6]. Latin America and Europe revealed the highest production of consumers’ wastes, which correspond to 200kg and 180kg per capita per year, respectively. The food loss in the regions of North America, Oceania, North Africa and West and Central Asia is estimated to reach 175kg of consumer waste per capita per year. Industrialized Asia, sub-Saharan Africa, and South and Southeast Asia are following, with food loss in the latter regions being estimated at 155kg, 150kg and 110kg of consumer waste per capita per year, respectively [7]. Central and Southern Asia, North America and Europe recorded the highest amounts of food loss for 2016 [5]. In countries which are characterized by high consumer incomes, food loss is 307 g per inhabitant per day, a value which is twice that of those with more modest incomes [6].

In industrialized countries, waste generation is recorded at later stages of the supply chain. Unlike industrialized countries, in the developing ones, wastes are generated at the initial stages of the food supply chain due to the lack of economic and technical capabilities during harvesting, storage and cooling procedures [8]. Therefore, waste management must be addressed by applying novel strategies which will contribute to food waste depletion with a parallel optimization of production procedures, targeting a transition from the linear model of consumption and production to the circular [1].

Food wastes definition includes all the discarded parts which are generated from food products, even those with enhanced nutritional profile [9]. These wastes can be considered as an appealing and economic source with a diversity in their nutritional composition, which varies according to their origin. They are composed of carbohydrates, proteins, lipids, and other significant ingredients. Their enhanced nutritional composition converts them to raw materials, which can find wide applications in numerous industries. Food by-products’ valorization could be adopted, for example, by the aroma industry, owing to the increased market demand for low-cost natural products [1].

Nowadays, the approach concerning the applied waste management strategies depends on the type of area. To be specific, in rural areas and farms the generated organic wastes are valorized either as animal feed or as humic compounds by applying composting procedure and then being used as fertilizers in fields. In urban areas, household wastes are either incinerated or disposed in open fields, exhibiting a negative environmental impact regarding air pollution and groundwater contamination [10].

Waste valorization perspective is associated with the feasible and sustainable methodologies which facilitate the procedures of recycling and reusing. Waste valorization strategies target the improvement of a product’s value by transforming wastes into alternative sources for the development of new high-added-value products. These developed products could be adopted by the global economy, contributing to the reduction of the environmental footprint since they obey the environmental standards [1]. 

The main objective of this review is to describe the valorization of generated wastes which are derived from the food chain in recent industrial applications. Their exploitation highlights a new pathway for the development of new high-added-value products, since they can be used as additives in food products (dairy, meat, sweets and baked foods, etc.), beverages and animal feed. This strategy is feasible and sustainable since their supplementation has the potential to upgrade the nutritional composition of the developed fortified products, with a parallel contribution to the reduction of the environmental footprint.

## 2. Feedstuffs

In recent years, a significant issue has emerged concerning the increased cost of feedstuffs, owing to the increased price of cereal grains and soybean products. Therefore, it is imperative to discover new pathways to alternative sources which can be used in the livestock sector with a simultaneous reduced cost. Agrifood by-products form an intriguing case, since they can be adopted by this field in order to develop novel feedstuffs [11]. Additionally, the aforementioned processing stages contribute to the reduction of the environmental footprint since the aforementioned by-products are discarded into the environment [12]. Therefore, the produced feedstuffs have the potential to improve the quality of several aspects of animal products. Table 1 describes the incorporation of agri-food by-products into feedstuffs.

Cows’ diets afforded a replacement up to 30% *w*/*w* with a mixture of ensiled tomato and apple pomace, resulting in a slight increase in milk yield without however provoking any negative effects on the animals’ health [13,14]. Additionally, the incorporation of a mixture of ensiled apple pomace and broiler litter (15 or 30% *w*/*w*) in dairy cows’ diets has proved to have beneficial effects on milk yield and dry matter intake [15].

Another study stated that the incorporation of a fermented apple supplement of 2% *w*/*w* amplified some significant parameters concerning finishing pigs, such as growth performance, daily and feed intake, feed efficiency and finally carcass weight [16]. Moreover, apple pomace addition of 10% or 20% *w*/*w* has the potential to promote beneficial bacteria as well as to diminish volatile fatty acids emissions, with the proportion of 10% *w*/*w* resulting in a more extensive nitrogen retention [17]. Apple pomace incorporation in piglets’ diets of 3.5% *w*/*w* has the ability to improve and enhance their good health and welfare, since it has proven beneficial effects on some crucial factors (gut microbiota, blood parameters) [18,19].

Laying hens’ fortified diets with apple pulp up to 10% *w*/*w* combined with 0.05% multienzyme supplement upgraded their performance, their egg traits and improved their blood parameters [14,20].

Citrus pulp was added to cows’ diets at a ratio of 1kg per day, exhibiting positive effects since their milk’s concentration of total polyphenols and flavonoids, as well as the milk’s antioxidant capacity, were found to be increased [21]. Lambs’ dietary incorporation of citrus pulp (30–45% *w*/*w*) [23,25] and orange pulp (10% *w*/*w*) [24] did not affect their feed intake, growth performance and carcass characteristics. On the other hand, the parameters of daily gain, feed intake and feed conversion were improved after the addition of dehydrated citrus pulp to the feedstuffs of growing kids [14,22]. According to recent studies, cereals’ substitution with dried citrus pulp in sheep’s diets (24–35% *w*/*w*) has the potential to contribute in a physical way to the improvement of meat oxidative and protein stability [26,27]. Concerning broilers, their feedstuff enrichment by dried citrus sinensis peel at a ratio of 1.5% or 3 % *w*/*w* did not affect their final weight, hot carcass weight and carcass yield despite the fact that thigh and breast weight were found to be reduced by 3% [28]. Nazok et al. [29] stated that the inclusion of 12% *w*/*w* of dried citrus pulp in laying hens’ diets did not influence the performance and egg quality in the early stage of production.

Pigs’ diets were enriched with tomato remnants of 3% or 5% *w*/*w*, resulting in a slight effect on the pork’s meat attributes, namely, its tenderness [30]. Tomato silage can be included at the level of 30% *w*/*w* of dry matter basis in fermentable liquid diets for growing-finishing pigs. It has been observed that this addition promotes their growth without exhibiting adverse effects on carcass parameters [31]. A fortified diet with 6% *w*/*w* of tomato pomace could enhance the acceptability of cooked meat from fattening rabbits [32].

Tomato by-products consist of a wide variety of bioactive substances, with lycopene forming the predominant compound. Lycopene is responsible for the tomato’s color and has been found to exhibit antioxidant, hypolipidemic and anticarcinogenic activities [43]. Lycopene’s addition to the lamb’s diet at 0.5, 0.1 or 0.2 g/kg resulted in higher dry matter intake and average daily gain. Moreover, lamb meat displayed a darker and redder color, which was observed by the lower lightness and hue angle values at 24 h. Lycopene’s addition contributed to the abatement of muscle fat concentration and lipid oxidation values, whilst the content of polyunsaturated fatty acids recorded an increase as compared to the control [34]. The inclusion of lycopene at 0.2 g/kg led to a better antioxidant profile since the content of total cholesterol, total triglycerides and lipoprotein cholesterol were found to be reduced, followed by a parallel increase in total antioxidant activity [14,33].

Laying hens’ diet was fortified with lycopene at the levels of 0.02, 0.04 or 0.08 g/kg. The obtained results showed it affected positively their fertilization rates and indicated an increased antioxidant activity and improved metabolism [35]. Furthermore, animals that were hydrated with lycopene-enriched water at 0.5 g/L recorded an improved serum bactericidal activity for broiler breeders compared to control groups [36]. An increased supplementation of lycopene at 0.75 g/kg to the broiler’s diet exhibited beneficial effects concerning growth performance characteristics and meat quality [37]. Additionally, lycopene’s inclusion in a broiler’s feedstuff at the level of 12 ppm has proved to have beneficial effects by contributing to cholesterol content reduction in thigh muscle [38]. It must be noted that the incorporation of lycopene at 0.5 g/kg did not affect broilers’ attributes of growth, slaughter performance or antioxidant enzymes in the breast meat, thigh meat, liver and kidney [39].

Filter cakes and dry potato peels are considered as perfect carbohydrate agents, justifying their valorization in the livestock field.

Corn’s replacement with potato wastes in cattle’s diets does not influence their metabolic state or milk status. Potato wastes are usually administrated in a dry and dehydrated form. However, the application of using potato wastes in a wet form has been investigated in order to reduce the drying cost. Wet potato wastes were supplemented in cattle’s diets up to 20% *w*/*w* without exhibiting adverse effects [40].

Carrot wastes were added to pigs’ diets at 20–25% *w*/*w*. The aforementioned residues are composed of 11.3 MJ/kg dry matter metabolizable energy, which justifies their exploitation as suitable animal feed as an additive in dairy cows’ diets [41].

Onodera et al. [42] developed silages which contained barley shochu by-product and pulps (juice residues) of Mandarin oranges and carrots. The developed silages presented enhanced nutritional composition since their crude protein and crude fiber content were upgraded. Additionally, moisture’s presence and nitrogen’s free extract values were increased.

The incorporation of dried carrot wastes up to 50% *w*/*w* revealed its potential to be used in growing rabbits’ diets as an alternate ingredient which can be administered without provoking negative impacts concerning the parameters of productive performance, nutrient digestibility, blood components and economic efficiency [41].

According to Table 1, the addition of apple and citrus by-products in cow’s diets presented beneficial effects on milk yield. More specifically, apple by-products addition up to 30% *w*/*w* led to a slight increase in milk yield without exhibiting any negative impact concerning their health, whereas citrus by-products incorporation of 1 kg enhanced their milk’s antioxidant capacity. Carrot wastes supplementation in a cow’s diet showed a different behavior compared to apple and citrus by-products, since its addition contributes to the elevation of metabolizable energy.

Additionally, the feedstuffs of finishing pigs were enriched with apple by-products in various proportions ranging from 2% up to 20% *w*/*w*. The results revealed that the various additions affected different parameters of their performance. To be specific, it was observed that the supplementation with 2% and 3.5 % *w*/*w* exhibited a positive impact on their growth and feed characteristics and promoted beneficial effects on gut microbiota and blood parameters. A common feature tomato by-products incorporation has with that of apples in finishing pigs’ diets is the improvement of their growth performance. The major difference between them is the ratio which is included in their diets, since according to Table 1 citrus was incorporated at a ratio of 30% *w*/*w*, in contrast to apple, which was incorporated at 2% *w*/*w*.

Apple, citrus and tomato by-products were incorporated into laying hens’ diets. The inclusion of both apple and citrus by-products at a similar ratio in their diets led to improved performance of egg traits and blood parameters. Tomato by-products incorporation revealed a different approach to their diet, since the extracted lycopene exhibited a positive impact on their fertilization rates, increased their antioxidant activity and improved their metabolism.

The common feature of lambs’ dietary fortification with citrus (30–45% *w*/*w*) and that with tomato (0.1–0.5 g/kg) by-products concerned the improvement of their feed intake by adding these by-products in various ratios.

According to Table 1, tomato by-products were used in more applications for broilers’ diets compared to those of citrus. The evaluation of the examined parameters concerning broilers revealed that the inclusion of tomato wastes in various proportions exhibited different positive effects on a broiler’s characteristics. Furthermore, citrus by-products were added at 1.5–3% *w*/*w* without exhibiting any negative impact.

As for rabbits’ diets, only tomato and carrot wastes were used in order to enrich their feedstuff. Tomato’s addition enhanced the acceptability of the cooked meat, whereas carrot’s addition did not present any negative effect on their performance and health. However, it must be noted that tomato’s addition did not exceed the level of 6% *w*/*w*, while carrot was included up to 50% *w*/*w*.

For sheep and cattle, only citrus and potato wastes were used in order to enhance their diets. Citrus addition ranged between 24–35% *w*/*w* in sheep’s diets, improving their meat oxidative and protein stability, whereas potato wastes and wet potato wastes were supplemented in cattle’s diets without revealing negative impact.

Overall, agri-food wastes found wide applications in the livestock sector as additives in animals’ diets, aiming at the improvement of feedstuffs quality and thus promoting the animals’ welfare. According to the present study, these wastes were incorporated in different ratios into animals’ diets, exhibiting a positive impact on the parameters that are related to their productivity and their health. Furthermore, the utilization of these by-products contributes to the reduction of production costs and the environmental footprint.

## 3. Food Applications 

### 3.1. Bakery Applications

#### 3.1.1. Bread 

Fruits and vegetables are considered as essential ingredients which should be included in a human’s diet. The produced by-products which are derived after their consumption/exploitation are characterized by their rich nutritional composition and the presence of a plethora of bioactive compounds, which converts them to valuable materials. The food industry has the potential to adopt them as natural additives by reaping their benefits for the development of many novel foods, in order to fulfill human nutritional needs.

Concerning baked products, apple pomace’s addition must be controlled and regulated at low levels since it has been found that its dietary fiber content impacts the rheological and pasting dough characteristics [44]. According to Bchir et al. [45], the parameters, namely, water absorption, stability and dough resistance to the extension, were improved after the enrichment with apple pomace of 2% *w*/*w*. Gumul et al. [46] examined the enrichment of wheat bread by adding whole and milled apple pomace at the levels of 5%, 10% and 15% *w*/*w*. The results revealed a higher moisture content regarding the crust, with the fortified bread by the whole pomace displaying a reduction in its volume. Baking loss was also recorded, especially for the enriched bread with 5% *w*/*w*. Moreover, whole pomace incorporation displayed a better phenolic profile, with the fortified bread with whole pomace at 5% *w*/*w* being the most acceptable to consumers due to its characteristics. The addition of 1% *w*/*w* of apple pomace to French bread had the potential to improve the sensory attributes [47]. Masoodi and Chauhan [48] investigated the replacement with 2%, 5%, 8% and 11% *w*/*w* of apple pomace in wheat bread’s formulation. The results showed that apple pomace addition between the levels of 0% to 11% *w*/*w* led to an increase in a loaf’s weight by 3.1% for the neutralized dough (dough with acidity-neutralized apple pomace), and by 7% for the un-neutralized one. Moreover, a loaf’s volume was decreased by 26.6% and 42.8% with neutralized and un-neutralized doughs, respectively. The increased dose of apple pomace incorporation contributed to an increase in crust color and bread hardness, with the dose of 5% *w*/*w* being the most acceptable. Jannati et al. [49] examined the quality of a traditional bread from Iran called Sangak bread when adding apple pomace powder (1% to 7% *w*/*w* of flour). The results indicated that the addition impacted the hardness of bread texture, since its values were reduced, and the staling process was found to be inhibited. The inclusion of 3% *w*/*w* was the most efficient, despite the fact that by incorporating less than 3% *w*/*w* the attributes including smell, texture and overall acceptability could be improved [48].

Mironeasa et al. [50] examined the addition of tomato seed flour in bread formulation in order to evaluate the improvement of several attributes. To be specific, wheat flour was substituted with tomato seed flour at the levels of 5%, 10%, 15% and 20% *w*/*w*. The addition upgraded some physical parameters like loaf volume, porosity and bread elasticity, with the addition of 10% *w*/*w* being the most impactful. The textural characteristics were also affected, with the bread’s hardness displaying an increase after the enhancement with tomato seed flour. Among all the physical parameters, the crust structure was especially affected by the increased levels of incorporated tomato seed flour. To be specific, the crust was found to be darker, compared to the bread samples with lesser additions, as well as with the control sample. Another study was conducted for the assessment of tomato seed flour’s impact on pan bread’s nutritional attributes. The obtained results revealed an increase concerning pan bread’s parameters of crude protein and crude fiber content as the levels of tomato seed flour were increased. The addition of 15% *w*/*w* was the most impactful for the parameters of proteins and crude fiber content, which were increased by 15.4% and 5.1%, respectively. On the contrary, the supplementation by 10% *w*/*w* gained the highest score for sensory analysis [51]. Tomato pomace was incorporated into bread and muffin formulation in order to replace refined flour at 35% and 40% *w*/*w*, respectively. Its incorporation has the potential to enrich the nutritional composition of the prepared products with bioactive compounds like dietary fiber, vitamin C and minerals, as well as to enhance their antioxidant activity. The fortified samples displayed an enhanced dietary fiber content and an increased concentration of vitamin C compared to the control. Furthermore, in the fortified samples the color attributes, namely, redness and yellowness, indicated an increase, while lightness values were decreased. The examined samples presented a softer texture compared to the control samples, as well as gaining consumers’ acceptance for their sensory attributes [52]. Tomato pomace contains a high content of hydrocolloids. Some of them have the potential to improve dough and bread quality. Majzoobi et al. [53] included tomato pomace powder in flatbread (Barbari bread) formulation using various concentrations (1–7% *w*/*w* flour basis). According to their results, tomato pomace was responsible for the improvement of the bread’s quality. Dried tomato wastes (skins and seeds) were incorporated in the bread’s formulation in order to enhance the presence of bioactive compounds [54]. The authors stated that the parameters of moisture content, titratable acidity and bread crust elasticity were upgraded in the fortified bread sample using tomato wastes. On the contrary, a reduction was observed in the volume and bread crust porosity of the examined sample. According to the sensory analysis, the fortified bread at 6% *w*/*w* led to good sensory attributes and gained overall acceptance.

Potato peel has acquired interest since its composition is characterized by the presence of dietary fiber, which is estimated at about 50% *w*/*w* [55]. Another study examined the physicochemical properties of potato peels and concluded that total dietary content, water holding capacity and the reduced amounts of starchy components are better than wheat bran’s. Wheat bread was prepared by replacing wheat flour with potato peels, resulting in an elevated crust darkening and a depleted loaf volume [55].

Orr et al. [56] reported that the addition of potato peels contributed to a musty odor in breads, but the applied process for potato peel extrusion prior to its use has the potential to reduce this aroma in the prepared products.

Whole-wheat bread formulation was developed by adding flour which is partly or entirely milled and derived from whole wheat grains. The prepared product can also be called brown bread. Carrot pomace was included in the recipe at 5%, 7.5% and 10% *w*/*w*, contributing to the enrichment of carotenoids, fiber and mineral compounds in the bread’s composition [57]. The physicochemical properties of the prepared bread did not reveal any negative effects, with the inclusion of 5% *w*/*w* recording an elevation of dough water absorption by 8% and bread volume by 10%. Furthermore, the bread’s crust was found to be elastic, gaining the highest scores [58].

Kumar and Kumar [58] developed buns, or small-sized wheat breads, by fortifying them with carrot pomace at various proportions (0%, 2.5%, 5%, 7.5% and 10% *w*/*w*). All the employed ingredients were mixed, contributing to the obtainment of a desirable dough consistency, followed by a baking process in a hot air oven at 177 ± 2 °C for up to 40min until a golden brown color. The parameters of expansion, water solubility, and absorption index were reduced as the ratio of pomace addition was increased, while the attributes of moisture and bulk density revealed an increase as the pomace incorporation was elevated. Carrot pomace fortification by 2.5% *w*/*w* exerted the best scores in the sensory assessment [41].

As shown in Table 2, apple by-products were incorporated in the manufacturing process of bread, mainly in the form of apple pomace. Apple pomace was supplemented up to 20% *w*/*w*, improving the physical and sensory attributes of the produced bread. The additions by 3% and 5% *w*/*w* were the most acceptable to consumers. Tomato wastes were incorporated in various ratios in bread formulation. Most applications incorporated tomato seed flour, pomace powder, etc., at up to 15% *w*/*w*, and highlighted the improvement in physical and sensory parameters, whereas when tomato pomace was included at 35–40% *w*/*w*, it presented a different behavior since it enriched the dietary fiber content and affected the color values. Carrot by-products were also used in bread preparation with the maximum addition reaching 10% *w*/*w* and resulting in the best scores in sensory acceptance. A common feature of all utilized by-products in bread formulation was the beneficial effects concerning the physical and sensory characteristics. Additionally, according to Table 2, the additions up to 10% *w*/*w* gained consumers’ acceptance by obtaining the best scores in the sensory evaluations.

#### 3.1.2. Sweet Bakery Products

Apple pomace has been widely used in the sweet bakery industry, aiming at the enhancement of flavor and nutritional values [49,59,60,79,80]. Apple pomace was added in cake formulation at various levels (5%, 10% and 15% *w*/*w*). It was observed that the increased addition of apple pomace led to a significant parallel reduction of the cake’s volume, with the particle size presenting an opposite behavior. The parameters of shrinkage and uniformity index recorded a noteworthy increase after apple pomace fortification, without significant differences between the additions of 10% and of 15% *w*/*w* [59]. Sudha et al. [60] produced cakes by fortifying them with apple pomace (up to 30% *w*/*w*) and stated that the manufacture of a cake by adding apple pomace at the level of 30% *w*/*w* reduced its volume by 37%, as compared to the control. With apple pomace additions from 10% to 30% *w*/*w*, a cake’s density displayed an increase, resulting in a harder texture. The fortified cakes were found to be acceptable to consumers after the application of organoleptic tests, since they obtained high scores. The addition of 30% *w*/*w* constitutes an exception since it recorded lower scores in its evaluation [60]. As apple pomace’s inclusion in a cake’s formulation was increased from 0% up to 15% *w*/*w*, a higher water absorption ability was observed. Furthermore, as its addition to the dough increased, the dough became stiffer. On the other hand, the reduction of the cake’s volume affected positively the density, which was found to be increased since apple fibers are well known for their strong water-binding abilities [60].

Apple pomace has found wide application by taking advantage of its ability to replace wheat flour in muffin production. Sudha et al. [61] described how muffin production fortification with less than 20% *w*/*w* of apple pomace had a positive impact concerning their shape since they were symmetrical. Also, the attributes of color, taste and texture were evaluated, gaining a high score [61]. However, an addition exceeding 20% *w*/*w* affected negatively the crust and crust’s color since they were modified from creamier yellow to brown. On the other hand, Wang and Thomas [62] reported that muffins’ fortification with apple pomace exceeding the ratio of 50% *w*/*w* increased their overall acceptability to 79.2%, as well as enhancing their nutritional profile concerning total dietary fiber, total phenolic content and antioxidant activity [61,62].

Lycopene extract was added at 3–5% *w*/*w* to cake and cookie products, enhancing their antioxidant activity, resulting in a better color and improving their sensory characteristics [63]. In another study, muffins’ fortification with potato peels at 25% *w*/*w* led to acceptable quality with a parallel enhanced resistance to compression [64].

The addition of apple wastes to the category of sweet bakery products presented various properties based on the amount of the inclusion. To be specific, the addition of up to 30% *w*/*w* in a cake’s formulation resulted in a reduced volume cake, whereas an addition that exceeded 50% *w*/*w* enriched the nutritional profile of the developed cake, thus increasing its acceptance. Moreover, the addition of tomato and potato by-products at cakes’ and muffins’ preparation stages did not exceed the ratio of 25% *w*/*w* for either of them, exerting beneficial effects on several parameters such as sensory characteristics and color of the developed products. A significant difference, which has been observed from Table 2, is that in order to gain consumers’ acceptance for apple-based applications, the addition has to exceed the amount of 50% *w*/*w*, compared to potato-based applications, for which the best attributes are achieved by including potato by-products at the level of 25% *w*/*w*.

#### 3.1.3. Brittle Bakery Products

Cookie doughs are characterized by their rich nutritional composition in sugars and lipids, exhibiting an ability to cover the bitter taste which could be a result of apple pomace addition [79]. Lauková et al. [65] examined the partial substitution of wheat flour by the addition of hydrated apple pomace powder during the cookie manufacturing process. The results indicated that with increasing levels of pomace addition from 0% to 15% *w*/*w*, the physical properties of volume, diameter and porosity were significantly reduced. Concerning the sensorial parameters, the fruity flavor of the cookies was elevated, whereas the grain taste decreased after the replacement. Despite the fact that the attributes that concern the overall satisfaction with the enriched cookies were decreased in the sensory evaluation, the acceptance of all treatments exceeded 90%. Another study by Jung et al. [66] investigated the development of cookies by incorporating apple pomace flour at 15% and 20% *w*/*w*. Its addition affected their color, since they became darker and redder. Apple pomace addition in cookies’ formulation exerts the ability to reduce the value of their glycemic index [67]. By fortifying them with apple pomace between the levels of 0% and 20% *w*/*w*, the glycemic index was found to be reduced [67]. Kaushal and Joshi [68] reported that cookies’ enrichment with apple pomace up to 30% *w*/*w* upgraded their quality, while in another study where cookies were fortified at 50% *w*/*w*, the sensory attributes remained unaffected. Furthermore, the gluten presence in cookies was significantly reduced with a simultaneous increase in apple pomace flour at 50% *w*/*w*, whilst dietary fiber, phenolics, flavonoids and antioxidant capacity were elevated [69].

Mir et al. [70] developed a gluten-free cracker which was manufactured with brown rice flour and was fortified with 3%, 6% or 9% *w*/*w* of apple pomace. Its incorporation led to a significant increase concerning the antioxidant activity, and the presence of chlorine and potassium were found to be elevated.

Alongi et al. [67] stated that the attributes of firmness and glycemic index in biscuits were diminished by the partial replacement of wheat flour with apple pomace in their formulation. It must be noted that supplementation of apple pomace powder which exceeds the levels of 10–15% *w*/*w* displayed negative effects concerning the biscuits’ acceptance, with the ratio of 5 % *w*/*w* forming the optimum addition which did not affect their quality.

Dried tomato pomace powder was included in soda crackers’ formulation, in order to replace the flour at 4%, 8% and 12% *w*/*w*. The manufactured crackers revealed higher crude protein concentration and total phenolic and mineral content. The fortified product also displayed an increased antioxidant activity. The developed product’s overall acceptability was similar to the control’s concerning the parameters of color, smell, crispiness and flavor. A replacement of wheat flour with tomato pomace powder which exceeds the levels of 12% *w*/*w* is not suggested because of the sensory evaluation [71].

Cookies were fortified with tomato pomace powder at five different proportions, with the incorporation up to 5% *w*/*w* gaining consumers’ acceptance [72].

Abd-El-Magied [64] examined the addition of potato peels at 5% and 10 % *w*/*w* in biscuit formulation substituting wheat flour. The obtained results presented a lower stack weight while retaining overall acceptance.

Cookies were investigated for supplementation with potato peels at 10% and 15% *w*/*w*. The prepared products were darker and harder, with a smaller diameter, compared to control samples [55]. 

Kumar and Kumar [73] fortified wheat-flour-based cookies with carrot pomace in order to enrich their dietary fiber content. Dried carrot pomace was included in the cookies’ formulation with its addition ranging between 0–9% *w*/*w*. The developed product recorded an increase concerning moisture content and hardness. As for the color attributes, namely, Hunter’s L* (light vs dark) and a* (red vs green color) values, they were found to be elevated with the increase in the incorporated carrot pomace in cookies, while the b* (yellow vs blue color) value remained unaffected. Regarding the sensorial attributes, the fortified cookies gained attention since their scores ranged between fair to very good, while the highest score was observed for the fortified cookies with 6% *w*/*w*.

Hernandez-Ortega et al. [74] investigated cookies’ supplementation with microwave or hot-dried carrot pomace powders, targeting the enrichment of their phytochemical content. Cookie production followed a traditional formulation by substituting wheat flour with 30% *w*/*w* for both of the carrot pomace powders. It was observed that after the fortification the total fiber content revealed a 3.7-fold increase, which corresponded to 7.4% of fiber content daily intake after the consumption of just one cookie. Similar behavior was observed regarding the presence of carotenoids and phenolic substances. The fortified cookies using the microwave-dried carrot pomace powder displayed the most significant content of β-carotene, epicatechin, gallic and ferulic acids.

The developed cookies with the addition of black carrot pomace at various levels ranging from 0–15% *w*/*w* in the flour exhibited an elevated fiber profile, which can possibly be associated with the enriched polyphenolic content and the antioxidant capacity, as well. Cookie fortification with 15% *w*/*w* of pomace was the most impactful, since the latter addition resulted in the highest levels of polyphenols and antioxidant activity [75].

Carrot pomace was added at 10%, 20% and 30% *w*/*w* to wheat flour for the development of sweet and sweet ‘n’ salty biscuits with enriched fiber content [76]. The aforementioned addition contributed to the elevation of the spread ratio for both types of biscuits. The addition of 20% *w*/*w* did not record significant differences concerning the mean scores of the sensory attributes, whereas the incorporation of 30% *w*/*w* presented a significant reduction in the latter score for both groups of biscuits [41].

Gayas et al. [77] studied the potential of carrot pomace powder incorporation in defatted soy flour biscuits, in order to evaluate the sensory parameters. Moisture remained practically stable, since a slight increase was observed after the fortification, whereas the parameters of fat, ash, protein, crude fiber and β-carotene were slightly depleted. The addition of up to 5% *w*/*w* of pomace was considered as optimum concerning the flavor’s acceptance, whereas the texture was reduced with no adverse effects.

According to the aforementioned studies, the exploitation of the food industry’s derived by-products has been widely used for the production of brittle bakery products, namely, cookies, crackers and biscuits, and they haveevaluated their effects on the prepared products. Cookies underwent several incorporations of the generated by-products into their formulation in various proportions. To be specific, apple by-products’ inclusion forms an intriguing case since the different incorporation rates (0 up to 30% *w*/*w*) affected different attributes of the prepared products. Additionally, apple, potato and carrot inclusions in cookies had some common effects on the latter. Despite the different incorporation rates, all the included by-products affected cookies’ color parameters, and a similar addition of apple and carrot wastes enhanced their antioxidant profile. Apple and tomato wastes’ supplementation at a similar ratio in crackers had a similar impact on the developed products by enhancing their antioxidant profile. The fortified biscuits with the aforementioned potato and carrot by-products revealed an increased overall acceptance, whereas the enriched biscuits with apple at 5–15% *w*/*w* reduced the glycemic index. Finally, it must be noted that only the fortification with apple-derived by-products resulted in a reduced glycemic index in the enriched cookies and biscuits.

#### 3.1.4. Other Bakery Products

The enrichment of wheat rolls by carrot pomace powder in their formulation revealed increased values of hydration attributes. Carrot pomace’s inclusion affected some dough farinographic attributes such as elevated water absorption, the dough’s development period and stability, and resulted in a reduced mixing tolerance index. Moreover, its addition affected several qualitative characteristics of the developed product, such as the reduced loaf and cambering. As for the sensory parameters, the fortified loaves with pomace powder up to 3% *w*/*w* gained higher acceptance [78]. 

#### 3.1.5. Meat Products 

Many endeavors have been conducted in respect of agrifood wastes’ valorization in meat products, targeting the improvement of their nutritional content. Table 3 highlights the fortification of meat products with the aforementioned by-products. 

According to Table 3, the most commonly examined meat products which were fortified with apple-derived by-products are mutton nuggets [84] and mutton goshtaba (a traditional Kashmiri meatball) [93], chicken sausages [94], chicken nuggets [82], and buffalo meat sausages and patties [81,95]. Younis and Ahmad [81] reported that the development of buffalo meat patties by replacing meat content with apple pomace (2% up to 8% *w*/*w*) led to a significant positive correlation with the parameters of fat, moisture and crude fiber content. Buffalo meat patties were enriched with apple pomace powder at various levels (2%, 4%, 6% and 8% *w*/*w*). Apple pomace has the ability to prevent fat loss by its effective oil-holding capacity. The inclusion of apple pomace in patties resulted in a significantly higher moisture and crude fiber content, contributing to a simultaneous increase in the firmness, toughness, hardness, gumminess and chewiness of the patties. Younis and Ahmad [81] reported that the addition of up to 6% *w*/*w* is considered as an ideal ratio for incorporation in buffalo meat patties, based on the sensory evaluation. The same pattern was observed regarding the attributes of cooking yield and thickness of the patties, as well as the texture characteristics, including firmness and toughness. Cohesiveness and springiness were negatively affected by the additions which exceeded the ratio of 6% *w*/*w*. The fortification of apple pomace in buffalo sausages at the same proportions led to a similar behavior [95].

Chicken products were also examined regarding their fortification with apple pomace. To be specific, meat substitution by 10% and 20% *w*/*w* in chicken patties led to reduced hardness [66]. A similar behavior concerning hardness was exhibited in the low-fat prepared chicken nuggets by performing a substitution from 8% to 12% *w*/*w* of pomace [82]. Choi et al. [83] studied the partial replacement of 5% and 10% of the pork fat in chicken sausages with 1% and 2% *w*/*w* of apple pomace fiber. The results displayed that the addition of 2% *w*/*w* led to less cooking loss, lower pH value and high hardness, compared to the control.

Huda et al. [84] examined the fortification of mutton nuggets with apple pomace at the levels of 5%, 10% and 15% *w*/*w*. The attributes which concern emulsion stability and rheological properties were improved as a result of the supplementation with dietary fiber. Moreover, cooking yield recorded an increase, pH values were reduced and, finally, hardness presented a major reduction as a result of the parallel increase in apple pomace addition. According to Huda et al. [84], the fortified mutton nuggets with apple pomace at 5% *w*/*w* obtained a higher score concerning consumers’ acceptance compared to the other two formulations.

Tomato wastes are characterized by the presence of a plethora of bioactive compounds in their composition, forming an ideal source for utilization by the food industry in meat products as food supplements. Tomato carotenoids have the potential to interact with the developed food products after their supplementation, thus upgrading their nutritional, sensory and functional attributes, either by direct addition or by an alternative such as active food packaging.

Domínguez et al. [96] investigated the addition of tomato extracts to various meat products in order to evaluate their potential as natural additive agents. Meat fat can be easily oxidized, with unsaturated fatty acids forming the most susceptible group. Many researchers have concluded that the inclusion of tomato by-product extracts upgrades the nutritional composition of the developed products. To be specific, their addition provides a better nutritional profile and decreases lipid oxidation, as well as presenting the potential to prolong the shelf-life of meat products. Moreover, they contribute to the improvement of their sensory attributes and their overall acceptance [97].

Four batches of traditional Spanish salchichόn were prepared after the fortification of the meat’s formulation by lyophilized tomato peels (6 and 12 g/kg). The manufactured product revealed significant differences concerning color and sensory parameters compared to the control, whereas regarding overall acceptance it displayed a good score. The employed method of incorporating the peels instead of applying lycopene extraction caused an abatement in lipid oxidation during the ripening and storge stages, ensuring a greater amount of lycopene for the consumer [85].

Tomato powder was added in a frankfurter recipe, displaying very promising results which are related to oxidation levels. To be specific, the prepared products recorded lower pH values, resulting in reduced microbial activity. The authors stated that tomato powder has the potential to act as a natural additive for prolonging a product’s shelf-life and reducing its nitrite content [86]. So et al. [88] investigated the lycopene presence, lipid oxidation, antioxidant capacity, color, and sensory parameters of an enriched frankfurter sausage with various dried tomato waste powders (1–5% *w*/*w*). The powders’ inclusion amplified chroma values, redness, yellowness and hue angle, whereas lightness was reduced. The powders’ addition at high levels led to better lipid oxidation, as well as elevated lycopene concentration, whilst it reduced the scavenging processes in the examined sample. Furthermore, the addition of the powder at 2% *w*/*w* in sausages is considered as optimum, since this obtained the highest scores in terms of taste, texture, appearance and overall acceptability [87].

Beef hamburgers were enhanced with an amount of lycopene added to ground meat, with the addition ranging from 1.5 to 6 g/100 g. Hence, a most healthy product was prepared, with enriched lycopene and fiber content derived from tomato residues [89]. Alves et al. [98] fortified minced chicken breast meat with tomato waste (0.3% *w*/*w*) under high pressure at 300, 600 and 800 MPa. The authors evaluated the lipid oxidation in the fortified mixture during the stage of its storage at 5 °C for 15 days. They applied thiobarbituric acid reactive substances (TBARS) to the fortified mixture, evidencing tomato wastes’ efficacy concerning protection from lipid oxidation in the pressurized samples. Furthermore, the employment of high-pressure procedures possesses secondary lipid oxidation, with tomato wastes having a strong protective activity against lipid oxidation in meats.

Yadav et al. [91] examined the inclusion of wheat bran with dried carrot pomace in chicken sausages, targeting the enhancement of its fiber presence, in order to evaluate their impact on the quality parameters of fresh and refrigerated chicken sausages. Chicken sausages are an emulsion produced mainly from meat products, which are widely consumed. The obtained results indicated that the prepared chicken sausages with an upgraded fiber content have the potential to be developed by the individual addition of wheat bran and dried carrot pomace at the levels of 3%, 6% and 9% *w*/*w*. The fortification by 6% *w*/*w* of wheat bran and dried carrot pomace led to a high score concerning acceptance, and the shelf life reached 15 days under refrigerated temperature.

Orange dietary fiber was incorporated in dry-fermented sausage in order to act as a fat replacer. The obtained results showed that its addition at 1,5% *w*/*w* improved the sausage’s sensory profile [92].

Vast applications have been performed for the production of novel meat products by adding food industry by-products. Apple’s incorporation at a similar proportion revealed a reduction of hardness in most of the prepared products, while its inclusion at lower levels presented an opposite behavior in chicken sausages, since hardness was increased. Tomato’s supplementation in Spanish salchichόn and Frankfurter sausage affected the color attributes. Citrus inclusion in the preparation stages of dry fermented sausage exhibited beneficial effects on the developed product, since its addition highlighted citrus’ potential to act as a fat replacer agent. The enriched meat products with apple, tomato and carrot wastes resulted in an increased fiber content. Additionally, it must be noted that the fortified products incorporating these wastes at low levels gained consumers’ acceptance. The ratio of 6% *w*/*w* is considered as the optimum for apple and carrot-based meat products.

#### 3.1.6. Other Food Applications

Agri-food wastes were used in many bakery and meat products in order to enhance their nutritional value. Besides their exploitation in the bakery and meat industries, these wastes have the potential to be used in other food applications for the development of functional products. Table 4 presents agri-food by-product incorporation in other food applications.

Apple pomace has the potential to be adopted by the dairy industry due to its physical properties as a natural stabilizer and texturizer since it forms an appealing source of dietary fiber and polyphenols which can be included in dairy products such as yogurt [100]. Apple pomace was adopted by Wang et al. [99] in order to investigate its potential activity as a natural stabilizer and texturizer for an inset-type yoghurt. Different levels of apple pomace (0.1%, 0.5% and 1% *w*/*w*) were incorporated into skim milk, and then were fermented with a combination of *Streptococcus thermophilus* and *Lactobacillus delbrueckii* subsp. bulgaricus at 42 °C. The obtained results revealed that the supplementation with 1% *w*/*w* of pomace recorded a significantly elevated onset pH and lessened time of gelation. All the examined yogurts presented improved consistency and cohesiveness after 28 days of storage. However, apple pomace has also been investigated regarding its ability to be used as a dairy component in the form of freeze-dried powder. The incorporation of this powder at the level of 1% *w*/*w* proved that the attribute of gelation pH was increased, whilst the fermentation time was reduced during yogurt preparation, resulting in the development of a more viscoelastic, consistent and firmer yogurt gel [100]. According to the same study, it was also reported that the fortification of stirred yogurt with apple pomace permitted a higher addition of pomace at 3% *w*/*w*, causing a significant reduction of syneresis with a simultaneous increase in viscosity, firmness and cohesiveness of the matrix during 28 days of cold storage process [100]. An incorporation of apple pomace flour at 3% *w*/*w* to a yogurt formulation has the potential to contribute to the better regulation of body weight control, and to diabetes prevention as well, due to the profound effects of its enhanced phytochemical profile, its valuable antioxidant capacity, as well as its sensorial and textural properties [101]. Wang et al. [99] stated that the composition of apple pomace, namely, dietary fiber and phytochemical compounds, is responsible for the enhancement of fortified yogurt drinks. Apple pomace manages to stabilize acidic dairy products such as yogurt due to tomethoxyl-rich pectin. The addition of higher levels of apple pomace in yogurt’s formulation affects the color characteristics, with the value of lightness being reduced. Also, pH values were lowered, and titratable acidity revealed a significant increase during the storage period. The attributes of firmness, consistency, cohesiveness and viscosity index were found to be elevated with a parallel increase in apple pomace addition.

Zayan et al. [102] investigated the potential incorporation of the isolated lycopene oil derived from tomato peel waste for butter replacement in processed cheese preparation. More specifically, lycopene oil was incorporated at various levels during the cheese’s preparation, with the developed products being compared with cheeses that were produced only with butter. The fortified cheese products indicated a higher lycopene content, antioxidant capacity, meltability and sensorial attributes, thus highlighting the significance of the replacement as a means for fat reduction in dairy products.

Abid et al. [103] examined the lycopene rich extract derived from tomato processing residues as a functional ingredient which could be used in applications providing the ability to prolong the shelf-life of a traditional Tunisian butter. The enriched butter’s storage conditions remained stable, which was a result of the enhanced antioxidant activity. Another study was conducted in order to evaluate the employment of lycopene in Jordanian traditional sheep butter as a natural antioxidant agent [104]. The results displayed lycopene’s positive impact concerning the stabilization of butter recipes.

Additionally, another study was carried out in order to examine the effects of carrot wastes in yogurt formulation. To be specific, carrot wastes’ beads were included in yogurt recipes at 2.5% and 5% *w*/*w* and the impact of their addition was evaluated during a storage period of 28 d at 4 °C. The enriched yogurt revealed promising results since it was observed that both additions contributed to the suggested daily intake of a significant β-carotene content. Microbiological and physicochemical attributes of the developed yogurts were retained during the storage period. Finally, the prepared yogurts exhibited an enhanced antioxidant activity, thus highlighting carrot wastes’ potential to fortify yogurts, resulting in high nutritional composition [105].

Citrus’ dietary fiber profile is characterized by the pectin presence, which has the potential to act as a stabilizer, emulsifier and thickening agent owing to its functional properties. Hence, its incorporation into food products like jams, jellies, marmalades and similar products will upgrade their nutritional value [123,124,125].

During the last decades, citrus dietary fiber has been used as a means for enhancing water holding capacity and viscosity, resulting in the abatement of syneresis during food processing [90]. Yogurt fortification with orange fibers, which are composed of a high content of pectin, provides the ability to stabilize casein’s presence by acting as a filler agent, which can be exemplified by pectin’s possible calcium sensitivity [106]. Citrus fibers have been widely used in a plethora of food products and, more specifically, in those whose nutritional composition contains significant volumes of fats and oils. Therefore, sweet orange fiber proved its ability to replace fat in ice cream preparation by decreasing it at a ratio of 70%, without exhibiting negative impacts concerning the parameters of color, odor and texture [107].

Apple waste’s addition was mainly focused on yogurt fortification. The specific by-products were incorporated at low levels which did not exceed 3% *w*/*w*. Their inclusion in its formulation enhanced yogurt’s nutritional properties with the increase in consistency, being considered as a common feature after 28 days of storage. The adoption of tomato wastes in order to enhance dairy products’ profile concerned the fortification of cheese and butter products. Their incorporation in these products at various levels improved the nutritional characteristics. A common feature after the fortification of both cheese and butters was the enhancement of their antioxidant capacity. The increased antioxidant capacity can be considered as a common characteristic which is enhanced in dairy products by the addition of apple, tomato and carrot by-products. According to previous studies, citrus addition to dairy products presented a diversity of effects, which depends on the developed products. More specifically, citrus acted as a filler agent and a fat replacer after its inclusion in formulations of yogurt and ice cream, respectively.

Apple pomace constitutes an ideal ingredient which can be incorporated into confectionery products for reaping the benefits of its rich nutritional and functional content, including pectin, as well as a plethora of flavor compounds [126]. Hussein et al. [108] prepared a jam whose formulation was based on fruit by-products, namely, apple pomace, carrot peels, banana peels and mandarin peels [108]. The apple pomace-based jam revealed a high phosphorus content, and the total phenolic and flavonoid presence was enhanced. Finally, this jam was the most acceptable to consumers due to its fruity flavor and attractive appearance.

Apple pomace has been evaluated concerning its potential to be included in extruded snack products. The results revealed that the nutritional composition of the enriched products was upgraded without however provoking negative effects on their physical properties [80,127].

Based on the fact that the majority of the developed extruded snacks are gluten-free, the attribute of their sensory acceptance has to be evaluated. Apple pomace’s addition to extruded snacks reduced most of their sensory characteristics, such as external appearance, chewiness and flavor. The latter modifications were more distinguishable as the levels of apple pomace’s addition ranged from 5% to 15% *w*/*w*. A similar behavior was observed concerning total product quality, but this remained within the acceptable limits [109]. Reis et al. [110] prepared extruded products, which were composed of rice and wheat semolina flour, by incorporating apple pomace at three different levels (10% up to 30% *w*/*w*) in order to evaluate their nutritional properties. Apple pomace’s addition at 30% *w*/*w* led to a reduction in nitrogen’s solubility index by 23%, displaying a low protein denaturation. Furthermore, the attributes which were associated with the antioxidant profile (total phenolic and flavonoid content) were found to be significantly increased.

According to Table 4, extruded products were fortified only with apple by-products. Their fortification presented a diversity of effects on the examined parameters. More specifically, the fortification up to 15% *w*/*w* for the preparation of extruded snacks exerted a negative impact on the sensory and quality attributes, whereas the addition of 30% *w*/*w* revealed an enhanced antioxidant profile.

Concerning the fortification of beverages with apple wastes, Bortolini et al. [111] incorporated apple pomace into cider production, targeting phenolic compounds’ recovery. The obtained results led to an upgraded antioxidant activity, and the cider’s sensory evaluation was improved. Apple pomace has also been used as an additive in the wine industry. Its dietary fiber has the ability to absorb tannins which are present in red wines, with the tart taste being neutralized by the pomace’s fortification, contributing thus to a better flavor, especially for those with a short aging period [112,113].

Citrus is responsible for the production of numerous soft drinks, alcoholic beverages and several food products with a favorable taste. Their by-products have the potential to serve as a flavoring means, in order to improve the attributes of aroma and taste in the developed foods [128,129]. Enriched products with cold-pressed orange oil like cookies, chocolate, caramels, licorice, jelly and chewing gum are considered as more attractive by consumers. The ratio of 0.01% (*v*/*v*) for juices and concentrated juices is considered as the most ideal addition, which contributes to a more favorable flavor and aroma [114].

Beverages, namely, cider and wine, were supplemented exclusively with apple by-products, aiming at the manufacture of more attractive products with enriched nutritional characteristics. The aforementioned addition exhibited significant effects on the developed beverages, which affected their antioxidant profile and their flavor. Additionally, citrus by-products were used for the fortification of juices, in order to enhance their nutritional attributes. This addition revealed citrus’ potential to act as a flavoring agent since it enhanced the attributes of aroma and flavor of the generated products. Overall, apple and citrus additions in beverages exhibited a common pattern concerning their activity on sensorial parameters, since these were found to be improved in all the developed products.

Many interesting studies have been conducted for the evaluation of the fortified oils using carotenoids, which are isolated from dry tomato waste. Nour et al. [115] used different vegetable oils as solvents for the extraction of oils with a high carotenoid profile, presenting better thermal and oxidative stability. The applied isolation of dry tomato residues in vegetable oil led to colored functional oils with strong antioxidant capacity when they are consumed as food additives. Three types of oils were used for the evaluation of low quality edible oils which were fortified with tomato by-products [116]. More specifically, after refined olive oils’ fortification with tomato peels, carotenoid presence as well as their bioavailability were found to be increased, displaying thus an alternative pathway for tomato wastes valorization. Tomato peel oleoresin extract, derived from tomato industrial residues, was applied as a dietary stabilizer agent, replacing the synthetic ones for refined olive oil and sunflower oils, in order to avoid oxidation reactions during their long-term storage [117].

Benakmoum et al. [116] added tomato peels to refined olive oil at 5% and 10% *w*/*w* in order to upgrade the β-carotene and lycopene content. Carotenoid presence was increased with a parallel increase in the tomato peel addition, whereas lycopene content was enhanced in the oil sample which was fortified with 10% *w*/*w* of tomato peel. Furthermore, the latter addition recorded an increased β-carotene content.

Robles-Ramírez et al. [118] assessed ethanol extract derived from tomato waste as a natural antioxidant means for canola oil oxidative stability. The concentration of peroxide, diene and panisidine in canola oil was found to be decreased compared to samples without antioxidant addition after their oxidative storage in an oven at 65 °C for 144 h.

Nour et al. [115] investigated the influence of carotenoids, which came from dry tomato residues, by applying various extraction procedures in order to evaluate the oxidative stability and some other attributes of ten different vegetable oils. Significant amounts of carotenoids were observed in the fortified vegetable oils with 5% *w*/*w* of tomato by-products compared to the control samples. Extra virgin olive oil displayed the highest solubility, followed by rice oil. The enrichment with carotenoids indicated an improvement concerning some oils’ oxidative stabilities, namely, unrefined corn oil, refined sunflower oil and peanut oil. On the other hand, tomato residues’ incorporation has the potential to increase peroxide presence, as well as to reduce the induction period of other fortified oils [87].

Soybean oil was examined after its supplementation by orange peel extract, displaying a higher oxidative inhibition compared to the synthetic one [130]. The isolated essential oil from bitter orange peel prevented the growth of inoculated *Staphylococcus aureus* in *Sardina pilchardus*. The applied essential oils contributed to an improvement by suppressing sardine rancidity, revealing the potential to serve as an inhibitor of foodborne pathogens by exhibiting antioxidant and antimicrobial properties [119].

Tomato by-products were used for the enrichment of several oil types such as vegetable, refined, edible and canola. The enrichment with tomato wastes contributed to the improvement of their carotenoid content, especially when that addition ranged between 5% and 10% *w*/*w*, whereas soybean oil exerted a higher oxidative inhibition after its fortification with citrus.

Pasta is usually produced by including only durum wheat semolina in its formulation. Vast endeavors have been conducted in order to upgrade its nutritional characteristics by incorporating other flours or ingredients into the recipe. Whole meal durum wheat spaghetti was fortified with tomato peel flour up to 15% [120]. The amount of dietary fiber, as well as carotenoids’ presence, were found to be increased in the fortified spaghetti sample compared to the control. However, the improvement of the product’s overall quality reveals the necessity for hydrocolloid’s addition. 

Mishra and Bhatt [121] produced pasta by replacing refined wheat flour with carrot pomace powder at the levels of 5%, 15% and 25% *w*/*w* in order to assess the physicochemical properties, textural characteristics and sensory attributes. The inclusion of carrot pomace powder contributed to the improvement of the pasta’s appearance by enhancing its color. However, the reduction of the texture score was indicative of the parallel increase in the pomace addition. The fortified pasta with 15% *w*/*w* of carrot pomace powder was considered as the optimum level, since it gained the highest acceptance based on its attractive appearance and improved taste and flavor. Gull et al. [122] also stated that the inclusion of millet flours and carrot pomace in pasta’s formulation contributed to the reduction of its cooking qualities.

Pasta products underwent a fortification by carrot wastes targeting the improvement of their nutritional parameters. As shown in Table 4, in the prepared pasta, carrot by-products were included in the preparation stages up to 25% *w*/*w*. The different incorporation rates affected various attributes of the developed products. More particularly, the supplementation up to 15% *w*/*w* resulted in increased dietary fiber content and carotenoid presence, whereas the inclusion up to 25% *w*/*w* improved pasta’s appearance. The addition of 15% *w*/*w* is considered as the optimum since it gained consumers’ acceptance.

## 4. Pharmaceutical and Cosmetic Applications

Besides agri-food by-products’ exploitation in the food industry as additives, they are widely used in both the pharmaceutical and cosmetic sectors due to their high content of bioactive compounds. Table 5 presents their adoption by these industries for the production of novel products.

Tomatoes contain a lot of bioactive compounds which have the potential to be incorporated into pharmaceutical products. Within this context, numerous studies have highlighted tomato’s potential contribution to a reduced risk of cardiovascular disease, thus revealing its potential for incorporation into pharmaceutical formulations.

Concha-Meyer et al. [131] examined the antithrombotic activity of extracts which were derived from various tomato pomace compositions (whole, seedless and seeds). The extracts were developed by applying an ultrasound-assisted process and by using water or ethanol/water (1:1) as solvent. The results were indicative concerning the sonication time and the applied solvent for the exhibition of antiplatelet aggregation activity. Sonication cycles contributed to the improvement of the activity in the seedless extracts since their composition is characterized by a high flavonoid content, which exhibits potential therapeutic effects against cardiovascular disease.

Carotenoids are considered as a significant group which contains many bioactive substances. They have the potential to act as potent means for enhancing insulin resistance in order to contribute to diabetes treatment, since it is a critical risk factor for the development of type 2 diabetes mellitus. Tenore et al. [132] manufactured a glucose drink by incorporating a dried tomato peel powder into the recipe. The prepared drink was administered to healthy human subjects in order to assess its impact on glycemic and insulinemic responses. The developed drink was found to exhibit a significant activity on postprandial glycemia through an insulin-saving mechanism, by affecting plasma content since the generated peaks were lower compared to the reference glucose solution.

Carotenoids’ exploitation in cosmetics secures the ability to prolong a product’s shelf-life, and they have the potential to exhibit skin protection properties against oxidative damage [135]. The major drawback is that their supplementation has the potential to present negative effects concerning the color and smell of the formulation, affecting the overall acceptance.

Costa et al. (2021) developed fortified microemulsions and macroemulsions with lycopene-enhanced extracts derived from tomato by-products. The results were promising since lycopene extracts gave a yellowish color and an unclear odor to recipes. It must be reported that the presence of an antioxidant substance elevated the overall acceptance of the developed cosmetic products.

A significant aspect concerning citrus by-products is their exploitation by the cosmetic industry. They consist of essential oils, pectins, flavonoids, carotenoids and citric acid, which can be used in cosmetic applications for skin, hair and nails, as antifungal and antibacterial lotions, as well as for soaps, perfumes and toiletries [125,128]. Atolani et al. [134] stated that the produced soap using *Citrus sinensis* seed oil can be successfully adopted by the cosmetic industry, exhibiting antiseptic attributes with remarkable properties like good solubility, foaming ability, texture, color, low free caustic alkali, antimicrobial activity, antioxidant potential, antiparasite activity and low cytotoxicity. It must be noted that major amounts of recovered essential oils, which are derived from other citrus by-products, are used in applications for the production of toilet soaps, perfumes, cosmetics and other home care products [136].

According to Table 5, agri-food by-products which are derived in the process stages of the respective industries reveal the potential for their exploitation as pharmaceuticals and cosmetics, taking advantage of their nutritional composition, since they consist of a plethora of bioactive compounds. Tomato by-products were used for the production of pharmaceutical products, upgrading their quality by exhibiting their beneficial effects, namely, therapeutic effects against cardiovascular disease. Furthermore, the cosmetic industry has adopted both tomato and citrus wastes for the development of novel cosmetic products with enhanced antimicrobial and antioxidant activity, which will improve their overall acceptability.

## 5. Incorporation Strategies of Agri-Food Wastes into Foods 

Food industry-derived by-products can be used as additives in vast applications in order to develop novel foods by taking advantage of their rich nutritional composition. To be specific, proteins, lipids, starch, vitamins, minerals, fibers and antioxidants which are present in by-products’ composition can be isolated from their original source by applying extraction strategies, or they can be valorized directly for the development of functional products. Nowadays, the technology associated with food industry applications has revealed a rapid development, promoting by-products exploitation for the preparation of innovative and functional products.

Bioactive compounds may be affected by degradation reactions after their exposure to light, high oxygen levels, moisture, certain pH values and heat. Due to this fact, industries must apply technologies targeting their protection so they can be used for the development of new products. Encapsulation is considered as an important technique which has been adopted by the food industry, since it reveals the potential to upgrade significant attributes of the developed products, such as texture, taste and color. Encapsulation can be defined as the procedure by which certain compounds are packed into food-grade wall material, either as separate fractions or as a mixture of different ingredients, securing the ability to produce capsules with different properties [137]. Additionally, encapsulation provides the ability to enhance the presence of bio-active compounds in the prepared functional products, thus promoting their health benefits.

Microencapsulation has the potential to protect bioactive substances from environmental and processing conditions. Spray-drying and freeze-drying are considered as the most common strategies which are employed by the food industry in order to recover valuable compounds.

Spray-drying is one of the most applied methodologies and is employed in order to encapsulate hydrophilic and hydrophobic ingredients. It is characterized as a low-cost and fast procedure with great potential for scaling up. This procedure performs a fast atomization of a solution, dispersion, or emulsion into a chamber at a high temperature (over 100 °C) in order to extract the sample in powder form [138].

Freeze-drying is defined as a drying procedure where the applied solvent and/or the suspension medium is crystallized at low temperature and subsequently is sublimated into the vapor phase from the solid form [139]. Freeze-drying is a significant procedure since it secures the ability to preserve the chemical profile and antioxidant activity in foods which are susceptible to heat [140]. The consumption of high energy levels is considered as the most important drawback of the freeze-drying process since considerable drying time is required [141,142]. 

It must be noted that a comparison between freeze-drying and spray-drying reveals that spray-drying is the application which is considered as a cheap and easy technique with less processing time, yet resulting in products of similar quality [142]. Furthermore, besides the above strategies, spray chilling, complex coacervation and emulsification are some additional processes which are adopted by industries, since these procedures exhibit the potential to protect and regulate the release of bioactive substances. Overall, these incorporation strategies must be further investigated in order to optimize their functions, resulting in the maximum production of novel products.

## 6. Conclusions

The growth of environmental pollution raises the necessity for the development of new, feasible and sustainable technologies targeting the exploitation of the generated by-products in various sectors (livestock, food and pharmaceutical industries, etc.). This review illustrates the rich nutritional composition of wastes which are generated by the food industry, highlighting their potential for the development of new products reaping their nutritional and functional benefits. This review summarized the exploitation of fruit and vegetable by-products as additives in the food industry for the production of new functional foods (dairy, confectionery, meat, pasta and bakery, etc.), as well as their use in the livestock field, and indicated very promising results. Moreover, this study highlighted agri-food wastes’ potential to be used by the pharmaceutical and cosmetic industries since they have been found to exhibit considerable biological activity. The utilization of agri-food wastes as alternative sources for the production of high-value-added products needs to be further investigated by the food and biotechnological industries. Future perspectives include: (a) the application of low-cost and suitable strategies; (b) the optimization of specific methodologies; (c) the development of novel food and pharmaceutical products enriched with bioactive compounds; and (d) the management of consumer awareness, which need to be addressed in order to evaluate the efficiency of agri-food by-products as potential sources of bioactive compounds.

## Figures and Tables

**Table 1:** Addition of agri-food by-products to feedstuffs.

By-Product	Animal	Addition	Concentration	Effects	Reference
Apple	Cows	Mixture of ensiled tomato and apple pomace	Up to 30% *w*/*w*	Slight increase in milk yield without negative effects on their health	[13,14]
		Mixture of ensiled apple pomace and broiler litter	15% or 30% *w*/*w*	Beneficial effects on milk yield and dry matter intake	[15]
	Finishing pigs	Fermented apple supplement	2% *w*/*w*	Improved growth and feed characteristics	[16]
		Apple pomace	10 or 20% *w*/*w*	Promotion of beneficial bacteria and reduced volatile fatty acids emissions	[17]
	Piglets	Apple pomace	3.5% *w*/*w*	Beneficial effects on gut microbiota and blood parameters	[18,19]
	Laying hens	Apple pulp and multienzyme supplement	Up to 10% and 0.05% *w*/*w*, respectively	Improved performance, egg traits and blood parameters	[14,20]
Citrus	Cows	Citrus pulp	1kg per day	Increased milk’s antioxidant profile	[21]
	Lambs	Citrus pulp and orange pulp	30–45% and 10% (*w*/*w*), respectively	No effect in their feed intake, growth performance and carcass characteristics	[14,22,23,24,25]
	Sheep	Dried citrus pulp	24–35% (*w*/*w*)	Improved meat oxidative and protein stability	[26,27]
	Broilers	Dried citrus sinensis peel	1.5% or 3 %(*w*/*w*)	The addition did not affect their final and hot carcass weight	[28]
	Laying hens	Dried citrus pulp	12% (*w*/*w*)	No negative effect concerning the performance and egg quality	[29]
Tomato	Pigs	Tomato residues	3% or 5% *w*/*w*	Slight effect in pork’s meat attributes	[30]
	Finishing pigs	Tomato silage	30% *w*/*w*	Promotion of their growth	[31]
	Fattening rabbits	Tomato pomace	6% *w*/*w*	Enhanced acceptability of cooked meat	[32]
	Lambs	Lycopene	0.5, 0.1 or 0.2 g/kg	Increased antioxidant profile (0.2g/kg)	[14,33,34]
	Laying hens	Lycopene	0.02, 0.04 or 0.08 g/kg	Increased antioxidant activity and improved metabolism	[35]
	Broilers	Lycopene-enriched water	0.5g/L	Improved serum bactericidal activity	[36]
		Lycopene	0.75g/kg	Beneficial effects concerning growth performance and meat quality	[37]
		Lycopene	12ppm	The addition reduced cholesterol content in thigh muscle	[38]
		Lycopene	0.5g/kg	No adverse effects of growth and slaughter performance	[39]
Potato	Cattle	Potato wastes	-	No effect in metabolic state or milk status	[40]
	Cattle	Wet potato wastes	Up to 20% *w*/*w*	No negative impact	[40]
Carrot	Pigs	Carrot wastes	20–25% *w*/*w*		[41].
	Cows	Carrot wastes	-	Increased dry matter metabolizable energy	[41].
	-	Silages of barley shochu by-product and pulps (juice residues) of Mandarin oranges and carrots	-	Εnhanced nutritional composition	[42]
	Rabbits	Dried carrot waste	Up to 50% *w*/*w*	No negative impact	[41]

**Table 2:** Addition of agri-food by-products to bakery applications.

Category	By-Product	Product	Addition	Concentration	Effects	Reference
**Bakery Products**	Apple	Wheat bread	Whole and milled apple pomace	5%, 10%, 15% *w*/*w*	Reduced volume, baking loss, enhanced phenolic profile, increased acceptability	[46]
		French bread	Apple pomace	1% *w*/*w*	Potential to improve the sensory attributes	[47]
		Wheat bread	Apple pomace	2%, 5%, 8% and 11% *w*/*w*	Increase in loaf’s weight and reduction of its volume	[48]
		Sangak bread (traditional Iranian bread)	Apple pomace powder	1% to 7% *w*/*w*	Diminished values of hardness of bread’s texture	[48,49]
	Tomato	Bread	Tomato seed flour	5%, 10%, 15% and 20% *w*/*w*	Improved physical parameters	[50]
		Pan bread	Tomato seed flour	Up to 15% *w*/*w*	Increased crude proteins and crude fiber content	[51]
		Bread and muffins	Tomato pomace	35% and 40% *w*/*w*	Enhanced dietary fiber content and increased concentration of vitamin C	[52]
		Flatbread (Barbari bread)	Tomato pomace powder	1–7% *w*/*w*	Improved bread’s quality	[53]
		Bread	Dried tomato waste (skins and seeds)	6% *w*/*w*	Upgraded attributes of moisture, titratable acidity and bread crust elasticity	[54]
	Potato	White bread	Potato peels	-	Increased crust darkening and depleted loaf volume	[55]
		Bread	Potato peels	-	Musty odor in breads which is reduced by the applied process for potato peel extrusion prior to its valorization	[56]
	Carrot	Brown bread	Carrot pomace	5%, 7.5% and 10% *w*/*w*	Enriched carotenoids, fiber and mineral compounds content	[58,57]
		Buns or small-sized wheat breads	Carrot pomace	0%, 2.5%, 5%, 7.5% and 10% *w*/*w*	Expansion, water solubility and absorption index were reduced, while moisture and bulk density revealed an increase	[41,58]
**Sweet Bakery Products**	Apple	Cake	Apple pomace	5%, 10% and 15% *w*/*w*	The increased addition of apple pomace led to a significant parallel reduction of cake’s volume	[59]
		Cake	Apple pomace	Up to 30% *w*/*w*	The partial addition from 10% to 30% increased cake’s density resulting in a harder texture	[60]
		Cake	Apple pomace	0% up to 15% *w*/*w*	Increased water absorption ability	[60]
		Muffins	Apple pomace	<20% *w*/*w*	High score in the evaluation of color, taste and texture	[61]
		Muffins	Apple pomace	>50% *w*/*w*	Increased acceptability, enriched nutritional profile	[62]
	Tomato	Cake and cookies	Lycopene extract	3–5% *w*/*w*	Enhanced antioxidant activity, improved color and sensory characteristics	[63]
	Potato	Muffins	Potato peels	25% *w*/*w*	Acceptable quality of the developed products	[64]
**Brittle Bakery Products**	Apple	Cookies	Hydrated apple pomace powder	0% up to 15% *w*/*w*	Fruity flavor was elevated, whereas the grain taste was decreased	[65]
		Cookies	Apple pomace	15% and 20% *w*/*w*	The addition affected their color	[66]
		Cookies	Apple pomace	0% to 20% *w*/*w*	Reduction of glycemic index	[67]
		Cookies	Apple pomace	Up to 30% *w*/*w*	Upgraded quality	[68]
		Cookies	Apple pomace	Up to 30% *w*/*w*	The sensory attributes remained unaffected, increased antioxidant capacity	[69]
		Gluten-free cracker	Apple pomace	3%, 6% or 9% *w*/*w*	Increased antioxidant activity and enchanced chlorine and potassium presence	[70]
		Biscuits	Apple pomace powder	5%, 10% and 15%	Firmness and glycemic index were reduced	[67]
	Tomato	Soda crackers	Dried tomato pomace powder	4%, 8% and 12% *w*/*w*	Increased crude protein concentration and antioxidant activity	[71]
		Cookies	Tomato pomace powder	Five different proportions	The incorporation up to 5% gained consumers’ acceptance	[72]
	Potato	Biscuits	Potato peels	5% and 10% *w*/*w*	The developed products displayed a lower stack weight by retaining the overall acceptance	[64]
		Cookies	Potato peels	10% and 15% *w*/*w*	The prepared products were darker and harder with a smaller diameter	[55]
	Carrot	Cookies	Carrot pomace	0% up to 9% *w*/*w*	Effects in moisture, hardness and color parameters	[73]
		Cookies	Microwave or hot dried carrot pomace powders	30% *w*/*w* (for either of carrot pomace powders)	Increased total fiber content and enriched carotenoids’ and phenolic substances’ profile	[74]
		Cookies	Black carrot pomace	0–15% *w*/*w*	Enhanced fiber presence, polyphenolic content and antioxidant capacity	[75]
		Sweet and sweet ‘n’ salty biscuits	Carrot pomace	10%, 20% and 30% *w*/*w*	Increased spread ratio for both types of biscuits	[41,76]
		Defatted soy flour biscuits	Carrot pomace powder	-	The parameters of fat, ash, protein, crude fiber, and β-carotene were slightly depleted	[77]
**Other Bakery products**		Wheat Rolls	Carrot pomace powder	-	Increased hydration attributes’ values as well as elevated water absorption and dough’s development period and stability	[78]

**Table 3:** Addition of agri-food by-products to meat products.

By-Product	Product	Addition	Concentration	Effects	Reference
**Apple**	Buffalo meat patties	Apple pomace	2%, 4%, 6% and 8% *w*/*w*	Increased nutritional, textural and sensory characteristics	[81]
	Chicken patties	Apple pomace	10% and 20% *w*/*w*	Reduced hardness	[66]
	Low-fat chicken nuggets	Apple pomace	8% to 12% *w*/*w*	Reduced hardness	[82]
	Chicken sausages	Apple pomace fiber	1% and 2% *w*/*w*	The addition by 2% led to less cooking loss, a lower pH value and high hardness	[83]
	Mutton nuggets	Apple pomace	5%, 10% and 15% *w*/*w*	Emulsion stability and rheological properties were improved	[84]
**Tomato**	Spanish salchichόn	Lyophilized tomato peels	6 and 12 g/kg	Significant differences concerning color and sensory parameters compared to the control	[85]
	Frankfurters	Tomato powder	-	Reduced microbial activity and nitrite content	[86]
	Frankfurter sausage	Dried tomato waste powder	1–5% *w*/*w*	The addition enhanced chroma values	[87,88]
	Beef hamburgers	Lycopene	1.5 to 6g/100g	Development of an enriched product in lycopene and fiber content	[89]
	Chickenbreast meat	Tomato waste	0.3% *w*/*w*	Efficient protection from lipid oxidation	[90]
**Carrot**	Chicken sausages	Wheat bran with dried carrot pomace	3%, 6% and 9% *w*/*w*	Upgraded fiber content	[91]
**Citrus**	Dry-fermented sausage	Orange dietary fiber	1.5% *w*/*w*	Improved sausage’s sensory profile	[92]

**Table 4:** Addition of agri-food by-products to other food applications.

Category	By-Product	Product	Addition	Concentration	Effects	Reference
Dairy Products	Apple	Inset-type yogurt	Fermented apple pomace by a mixture of *Streptococcus thermophilus* and *Lactobacillus delbrueckii* subsp. bulgaricus	0.1%, 0.5% and 1% *w*/*w*	Improved consistency and cohesiveness after 28 days of storage procedure	[99]
		Stirred yogurt	Apple pomace	3% *w*/*w*	Significant reduction of syneresis followed by a simultaneous increase in viscosity, firmness and cohesiveness of the matrix	[100]
		Yogurt	Apple pomace	3% *w*/*w*	Regulation of a better body weight and diabetes prevention	[101]
	Tomato	Cheese	Lycopene oil	Various levels	Higher lycopene content, antioxidant capacity, meltability and sensorial attributes	[102]
		Tunisian butter	Lycopene rich extract	-	Stable storage conditions and enhanced antioxidant activity	[103]
		Jordanian traditional sheep butter	Lycopene	-	The addition displayed lycopene’s positive impact concerning the stabilization of butter recipes	[104]
	Carrot	Yogurt	Carrot beads	2.5% and 5% *w*/*w*	Enhanced antioxidant activity	[105]
	Citrus	Yogurt	Pectin-rich orange fiber	-	Stabilization of casein	[106]
		Ice cream	Sweet orange fiber	-	No adverse effects in color, odor and texture	[107]
Confectionery Products	Apple	Jam	Apple pomace	-	Enriched phospurus content and enhanced total phenolic and flavonoid presence	[108]
Extruded Food Products	Apple	Extruded snacks	Apple pomace	5% up to 15% *w*/*w*	Sensory characteristics and product’s quality reduction	[109]
		Extruded products	Apple pomace	10% up to 30% *w*/*w*	Increased antioxidant profile	[110]
Beverages	Apple	Cider	Apple pomace	-	Upgraded antioxidant activity and improved sensory evaluation	[111]
		Wine	Apple pomace	-	Improved flavor	[112,113]
	Juices and concentrated juices	Cold-pressed orange oil		0.01 *v/v*	Better flavor and aroma	[114]
Oil products	Tomato	Vegetable oil	Dry tomato residues	-	Colored functional oils with strong antioxidant capacity	[115]
		Edible oils	Tomato peels	-	Increased carotenoid presence as well as enhanced bioavailability	[116]
		Refined olive oil and sunflower oils	Tomato peel oleoresin extract	-	Avoidance of oxidation reactions during long-term storage	[117]
		Refined olive oil	Tomato peels	5% and 10% *w*/*w*	Increased carotenoid, lycopene and β-carotene profile	[116]
		Canola oil	Ethanol extract	-	Reduced concentration of peroxide, diene and panisidine	[118]
		Vegetable oils	Carotenoids	-	Carotenoid presence was enhanced by the addition of 5%	[115]
	Citrus	Soybean oil	Bitter orange peel extract	-	The addition prevented the growth of inoculated *Staphylococcus aureus* in *Sardina pilchardus* as well as improved the suppression of sardine rancidity	[119]
Pasta products	Carrot	Whole meal durum wheat spaghetti	Tomato peel flour	Up to 15% *w*/*w*	Increased dietary fiber content and carotenoid presence	[120]
		Pasta	Carrot pomace powder	5%, 15% and 25%	Improved pasta’s appearance by enhancing its color, reduced texture score with the parallel increase of pomace addition	[121]
		Pasta	Millet flours & Carrot pomace	-	Reduced cooking quality	[122]

**Table 5:** Addition of agri-food by-products to pharmaceutical and cosmetic applications.

Category	By-Product	Product	Addition	Concentration	Effects	Reference
**Pharmaceutical products**	Tomato-		Various tomato pomace compositions (whole, seedless and seeds)	-	Antiplatelet aggregation activity was improved by sonication cycles in the seedless extracts	[131]
		Glucose drink	Dried tomato peel powder	-	Significant activity to postprandial glycemia	[132]
**Cosmetics**		Micro and macro -emulsions	Lycopene enhanced extracts	-	Lycopene exhibited a yellowish color and unclear odor in recipes as well as elevated the overall acceptance	[133]
	Citrus	Soap	*Citrus sinensis* seed oil	-	Increased antimicrobial, antioxidant and anti-parasite activities	[134]

## Data Availability

Not applicable.
